# Contributions of early-life cognitive reserve and late-life leisure activity to successful and pathological cognitive aging

**DOI:** 10.1186/s12877-022-03530-5

**Published:** 2022-11-01

**Authors:** Yiru Yang, Yaojing Chen, Caishui Yang, Kewei Chen, Xin Li, Zhanjun Zhang

**Affiliations:** 1 State Key Laboratory of Cognitive Neuroscience and Learning, Beijing Normal University, No.19, Xinjiekouwai Street, Beijing, 100875 China; 2 School of Nursing and Rehabilitation, Cheeloo College of Medicine, Shandong University, Jinan, 250012 Shandong China; 3 Beijing Aging Brain Rejuvenation Initiative (BABRI) Centre, Beijing Normal University, Beijing, 100875 China; 4 School of Systems Science, Beijing Normal University, Beijing, 100875 China; 5 Banner Alzheimer’s Institute, Phoenix, AZ 85006 USA

**Keywords:** Successful cognitive aging, Mild cognitive impairment, Cognitive reserve, Leisure activity

## Abstract

**Background:**

The identification of factors that specifically influence pathological and successful cognitive aging is a prerequisite for implementing disease prevention and promoting successful aging. However, multi-domain behavioral factors that characterize the difference between successful and pathological cognitive aging are not clear yet.

**Methods:**

A group of community-dwelling older adults (*N* = 1347, aged 70-88 years) in Beijing was recruited in this cross-sectional study, and a sub-cohort was further divided into successful cognitive aging (SCA, *N* = 154), mild cognitive impairment (MCI, *N* = 256), and cognitively normal control (CNC, *N* = 173) groups. Analyses of variance, regression models with the Shapley value algorithm, and structural equation model (SEM) analyses were conducted to determine specific influencing factors and to evaluate their relative importance and interacting relationships in altering cognitive performance.

**Results:**

We found that abundant early-life cognitive reserve (ECR, including the level of education and occupational attainment) and reduced late-life leisure activity (LLA, including mental, physical, and social activities) were distinct characteristics of SCA and MCI, respectively. The level of education, age, mental activity, and occupational attainment were the top four important factors that explained 31.6% of cognitive variability. By SEM analyses, we firstly found that LLA partially mediated the relationship between ECR and cognition; and further multi-group SEM analyses showed ECR played a more direct role in the SCA group than in the MCI group: in the SCA group, only the direct effect of ECR on cognition was significant, and in the MCI group, direct effects between ECR, LLA and cognition were all significant.

**Conclusions:**

Results of this large-sample community-based study suggest it is important for older adults to have an abundant ECR for SCA, and to keep a high level of LLA to prevent cognitive impairment. This study clarifies the important rankings of behavioral characteristics of cognitive aging, and the relationship that ECR has a long-lasting effect on LLA and finally on cognition, providing efficient guidance for older adults to improve their cognitive function and new evidence to explain the heterogeneity of cognitive aging.

**Supplementary Information:**

The online version contains supplementary material available at 10.1186/s12877-022-03530-5.

## Background

The way we age is mainly decided by the quality of early-life development, the degree of middle-life maintenance, and the pace of late-life degeneration; therefore, aging heterogeneity is substantially influenced by various factors in lifespan experiences [1]. This heterogeneity is remarkably observed in cognitive aging: compared with steady cognitive changes in normal aging, accelerated cognitive decline is usually a characteristic of pathological cognitive aging in the form of mild cognitive impairment (MCI) and eventually dementia [2]; on the other end of the spectrum, older adults maintaining superior cognitive performance compared with their peers are commonly referred to as exhibiting successful cognitive aging (SCA) [3]. Prior studies used various definitions of SCA, including top memory performers [4] defined using cross-sectional data, successful cognitive ager [5] and memory maintainers [6, 7] defined using longitudinal data, and superager [8, 9] defined by generational comparison (older adults vs. young or middle-aged adults). How to define SCA remains debated, but well-preserved age-sensitive cognitive functions, like episodic memory and executive function, are often-measured domains. Since pathological and successful cognitive aging are located at opposite ends of the cognitive aging spectrum, studies aiming to identify how they are influenced by multiple factors differently are very important for both disease prevention and the promotion of successful aging [10].

Factors that influence cognitive aging heterogeneity and distinguish pathological and successful cognitive aging have been reported previously, although not as extensively. In addition to advanced age, the female sex represents a strong risk factor for Alzheimer’s disease [11]. In contrast, a longitudinal study of episodic memory found that the female sex was a predictor of membership in the maintainer group [6]. High educational attainment is one of the most commonly identified variables associated with SCA [12], but studies have yielded mixed results on its effect on cognitive decline [13]. Poor physical and mental health conditions were recognized as influencing factors of pathological cognitive aging [14, 15]; the issue of whether intact health conditions positively affect SCA remains unresolved [16, 17]. The maintenance of abundant leisure activities was shown to be beneficial to cognitive function [18, 19]. In addition, multiple aspects of the geriatric lifestyle are potential influencing factors, such as diet [20] and sleep quality [21]. From the lifespan perspective, the aforementioned factors are divided into two types. Factors throughout early and middle life, including educational and occupational attainments, etc., are usually nonmodifiable for older adults. Nevertheless, these factors have close relationships with cognition as cognitive reserve proxies [22, 23]. Certainly, many late-life modifiable factors have been identified, including leisure activities and daily lifestyle, etc. in old age [24]. More systematic investigations are needed to identify the specific roles of these factors in cognitive aging heterogeneity.

Although previous studies have provided lots of reference information on the factors influencing cognitive aging, several core issues still require further investigation. First, the specific or common effects of these multiple factors on pathological and successful cognitive aging are unclear. Factors influencing pathological cognitive aging have been reported and related prevention measures have been recommended to decrease the risk of related diseases, such as dementia [24]. In contrast, factors influencing SCA are relatively unconfirmed, and the degree of overlap with factors influencing pathological cognitive aging is not sufficiently depicted [16, 25]. Second, the relative importance rankings of these multiple factors in explaining cognitive heterogeneity are still largely unknown. Finally, the aforementioned early-life and late-life factors both exert direct effects on cognition; nevertheless, it’s not clear whether the relationship between them further explains their effects on cognitive aging, especially for factors with a high rank of importance in predicting cognitive heterogeneity.

In the current study, we sought first to identify influencing factors specific or common to successful and pathological cognitive aging by comparing the multidomain characteristics of SCA, MCI (typical representation of pathological cognitive aging), and cognitively normal control (CNC) groups. We then evaluated the relative importance of the identified factors to cognitive heterogeneity. Finally, we examined the relationships between early-life factors, late-life factors, and cognitive function. We hypothesized that SCA and MCI had both overlapping and specific influencing factors, and the overlapping factors would be more important in explaining cognitive heterogeneity. We also hypothesized the existence of a mediation effect of late-life factors on the relationship between early-life factors and cognition in addition to their detectable direct effects on cognition.

## Methods

### Participants

**Fig. 1:**
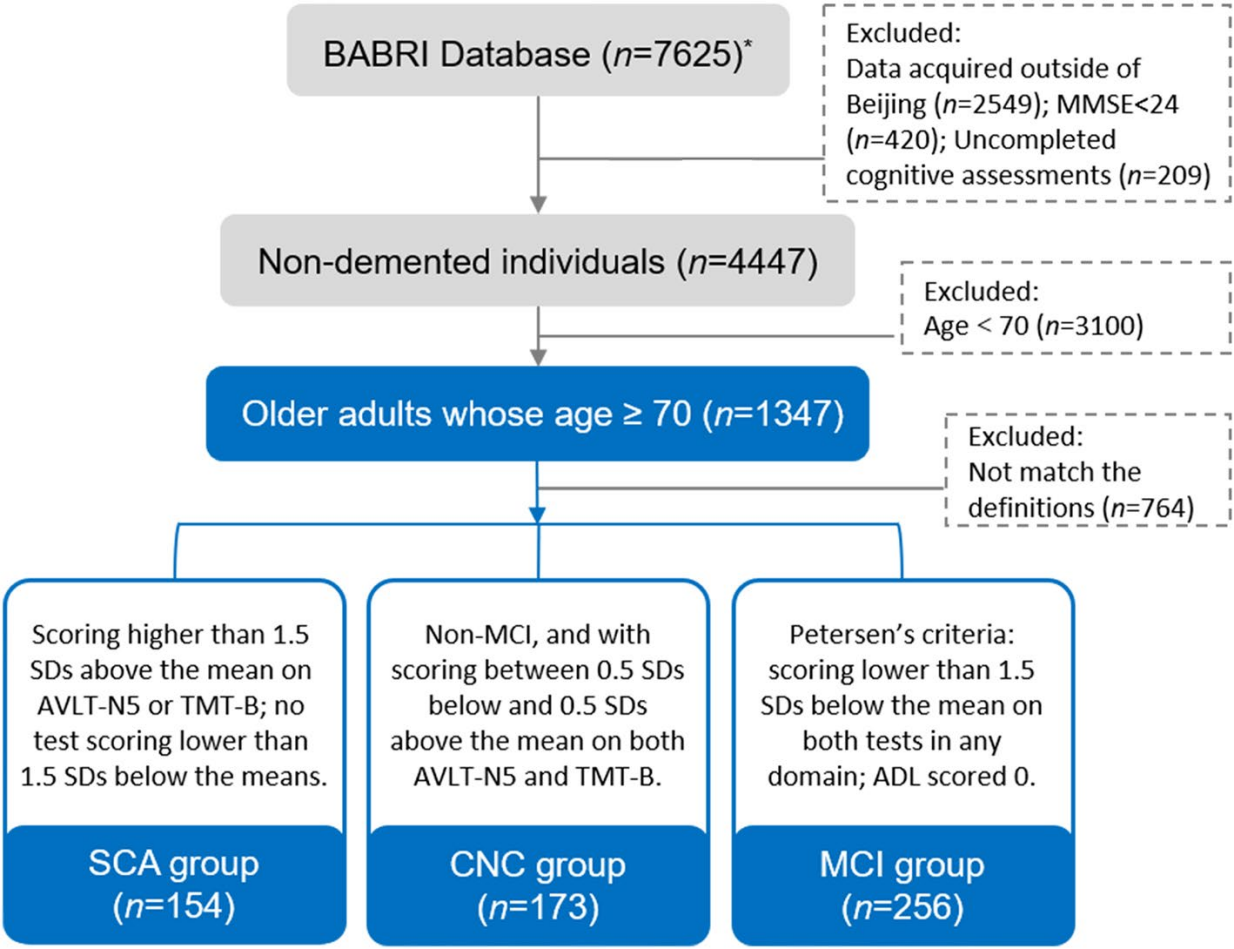
Flow chart of the participant inclusion process. ^*^Total data volume of the BABRI database (*n* = 7625) shown in this flowchart is up to 2018.1, the data volume is increasing since then and BABRI has now recruited over 10,000 participants [26]. Abbreviation: MMSE, Mini-Mental State Examination; AVLT-N5, Auditory verbal learning test long-time delayed recall; TMT-B, Trail-Making Test Part B; SD, standard deviation; ADL, activities of daily living scale

All participants were from the Beijing Aging Brain Rejuvenation Initiative (BABRI), an ongoing cohort study investigating aging and cognitive impairment in China [26]. The inclusion criteria for this study were as follows: (1) aged 70 years or older, (2) a score of at least 24 on the Chinese version of the Mini-Mental State Examination (MMSE) [27], (3) no history of severe neurological and psychiatric conditions, (4) no history of taking psychoactive medications, and (5) completed a battery of neuropsychological assessments. Among 7625 participants from the BABRI baseline database, 1347 non-demented older adults whose ages were larger than 70 (i.e., the full sample) were included following the criteria above in the current study, and these participants were further assigned to different groups (SCA, MCI, CNC) according to the definitions described below (shown in Fig. 1).

### Neuropsychological tests and grouping methods

Cognitive assessments covered multiple domains, including memory, attention, visuospatial ability, and executive function, and each domain was evaluated by two tests, as implemented previously [28]. The operationalized definition of SCA in this study was (1) excellent episodic memory or executive function, i.e., scoring higher than 1.5 standard deviations (SDs) above the age- and education-adjusted mean on the Auditory Verbal Learning Test long-time delayed recall (AVLT-N5) or the Trail-Making Test part B (TMT-B); and (2) intact general cognition, as evidenced the lack of a score on any test lower than 1.5 SDs below the age- and education-adjusted means. The diagnostic criteria for MCI were based on Petersen’s criteria [29]: (1) subjective cognitive complaints; (2) cognitive impairment in at least one domain, i.e., scoring lower than 1.5 SDs below the age- and education-adjusted mean on both tests in any domain and (3) the activities of daily living (ADL) [30] scored 0. The CNC group was defined as non-MCI and scored between 0.5 SDs below and 0.5 SDs above the mean on both AVLT-N5 and TMT-B. Based on the aforementioned definitions, the grouped sample (*N* = 583) included 154 participants with SCA (age range 70-88 years, mean age 73.84 ± 3.78 years, 100 females), 173 CNC participants (age range 70-86 years, mean age 74.80 ± 3.89 years, 88 females) and 256 participants with MCI (age range 70-88 years, mean age 76.11 ± 4.15 years, 133 females).

How to best distinguish supernormal cognitive maintenance in aging remains a controversial topic, and there are numerous operational definitions of SCA in previous studies [25]. In the current study, we paid attention to older adults whose ages were equal to or above 70, by which older adults have experienced the main process of age-related cognitive decline according to cognitive aging trajectory studies [31]; and those who still maintain superior cognitive performance at age 70 and above are more likely to be members of the SCA group than those under the age of 70. Regarding cognitive domains, episodic memory and executive function are given priority in the current study while defining SCA. Episodic memory and executive function are both sensitive to age-related decline [32] and maintaining superior performance in these two domains implies little age-related cognitive decline. The rationality of using these measures (AVLT-N5 and TMT-B for episodic memory and executive function, separately) is also recognized by prior work [5, 33], Moreover, no impaired domain was allowed in our SCA definition.

### Collection of behavioral characteristics

Detailed demographic information, mental and physical health conditions, living habits, and leisure activities were collected in one-to-one interviews between trained testers and participants. Demographic information included age, gender, level of education, occupational attainment, marriage status, and income level. The level of education was confirmed as years of formal schooling at an early age; and occupational attainment was measured by the occupational prestige scale (range 0-1) which represents the degree of occupational prestige and social status [34, 35]. Marriage status was divided into two types, married and single (for various reasons, including unmarried, widowed, and divorced); the income level was scored from one to thirteen, and a higher score indicated more monthly income for the participant. Surveys on mental health conditions included life self-satisfaction, memory self-satisfaction, loneliness, and depression. Life self-satisfaction was measured using the face scale, which included seven stick figures of faces that expressed increasing degrees of happiness from “extremely sad” to “extremely happy”; memory self-satisfaction was measured using the hill scale, in which older adults selected a step from 11 steps to represent their degree of satisfaction with their current memory function; Loneliness and depression were tested using the UCLA loneliness scale [36] and the Geriatric Depression Scale (GDS) [37], respectively. Physical health conditions were measured both subjectively and objectively. The subjective health status was reported by participants using a four-point rating scale (“good”, “normal”, “bad” or “poor”), and objective measures were collected including body mass index (BMI) and histories of multiple diseases, including diabetes, hypertension, hyperlipidemia, cerebrovascular disease, and coronary heart disease (all were marked as “0” for healthy individuals and “1” for patients). Living habits included diet, sleep quality, and histories of smoking and drinking. The Pittsburgh Sleep Quality Index (PSQI) scale [38] was used. Finally, leisure activity was measured using a scale with 23 items that were recognized as common activities in the older Chinese population. We used the weighted grade method to calculate scores for mental, physical, and social activities, as implemented previously [28, 39, 40].

### Statistical analysis

A three-step procedure was conducted as follows. First, we evaluated intergroup differences (SCA, CNC, MCI) in cognitive performance and behavioral characteristics. ANOVA, *χ*^*2*^, and Kruskal-Wallis tests were performed for continuous, categorical, and ordinal dependent variables, respectively, using SPSS 24.0 (IBM Corp, Armonk, NY), and factors with significant intergroup differences were identified. Secondly, we examined the significance of these identified factors in predicting cognitive performance (calculated as the average Z-score of each cognitive domain) using multivariable regression analyses. We then determined the relative importance of each factor by calculating the “lmg” metric using the “relaimpo” package [41] of R (version 4.0.3) to decompose the explained *R*^2^ of the regression model into nonnegative contributions of factors that automatically sum to the total *R*^2^ in the form of percentages. The Shapley value algorithm [42] was used to obtain the relative importance of each factor (see the equations below for the “lmg” metric, which is the average increase in *R*^2^ attributed to each factor).

1 $$\textrm{seq}{R}^2\left(\left\{{\textrm{x}}_{\textrm{k}}\right\}|{\textrm{S}}_{\textrm{k}}\left(\textrm{r}\right)\right)={R}^2\left(\left\{{\textrm{x}}_{\textrm{k}}\right\}\cup {\textrm{S}}_{\textrm{k}}\left(\textrm{r}\right)\right)-{R}^2\left({\textrm{S}}_{\textrm{k}}\left(\textrm{r}\right)\right)$$

2 $$\textrm{LMG}\left({\textrm{x}}_{\textrm{k}}\right)=\frac{1}{\textrm{p}!}\sum_{{\textrm{S}}_{\textrm{k}}\subseteq \left\{{\textrm{x}}_1,\dots, {\textrm{x}}_{\textrm{p}}\right\}\setminus \left\{{\textrm{x}}_{\textrm{k}}\right\}}\textrm{n}\left({\textrm{S}}_{\textrm{k}}\left(\textrm{r}\right)\right)!\left(\textrm{p}-\textrm{n}\left({\textrm{S}}_{\textrm{k}}\left(\textrm{r}\right)\right)-1\right)!\textrm{seq}{R}^2\left(\left\{{\textrm{x}}_{\textrm{k}}\right\}|{\textrm{S}}_{\textrm{k}}\left(\textrm{r}\right)\right)$$

The order of regressors in the regression model was determined based on all possible permutations of the available regressors (i.e., identified influencing factors) x_1_ … x_p_, and the order was denoted by the tuple of indices r = (r_1_ … r_p_). The denominator p! was the number of different permutations of all regressors, S_k_ denoted the set of regressors extracted from all regressors except for x_k_, S_k_(r) denoted the set of regressors entered into the model before the regressor x_k_ in the order r, and the number of permutations when the regressor x_k_ in the order r was counted as n(S_k_(r)) ! (p − n(S_k_(r)) − 1)!. The increase in *R*^2^ when adding x_k_ to the model with the regressors in set S_k_(r) was calculated as seq*R*^2^({x_k_}| S_k_(r)) in Eq. (1). In this study, *p* = 9.

In the third step, the SEM approach was used to test the proposed hypotheses of the relationships among early-life factors, late-life factors, and cognitive performance. The observed variables were first standardized and then put into the model. The root mean square error of approximation (RMSEA), comparative fit index (CFI), and Tucker-Lewis index (TLI) were used to assess the goodness of fit in SEM [43]. In addition, we examined the significance of factor loadings, path coefficients (*β*), and mediating effects using a bias-corrected bootstrap 95% confidence interval (CI) generated after a bootstrap analysis that resampled the data 1000 times. We first conducted SEM analyses with the grouped sample (*N* = 583) and supplementary analyses with the full sample (*N* = 1347) and then built models of the three groups separately to determine any differences. SEM analyses were conducted using Mplus V.8.3 software (www.statmodel.com).

## Results

### Multifactorial between-group differences

Between-group differences in cognitive performance and behavioral characteristics are summarized in Table 1 and Table S1. Sequential cognitive performances in all domains were observed in the order of best performance in SCA and worst in MCI, with CNC in between. The SCA group was younger than the CNC group, and both groups were younger than the MCI group. The SCA group had a larger proportion of women, higher levels of educational and occupational attainment, higher levels of memory self-satisfaction, and was more frequently involved in mental activities than the other two groups. In addition, the SCA group had a lower BMI than the CNC group. There was no significant difference between CNC and MCI in gender, level of education, occupational attainment, and BMI. The MCI group had worse memory self-satisfaction and participated in leisure activities less frequently than the other two groups, and there was no significant difference between SCA and CNC in physical and social activities. Taken together, we found that age, gender, level of education, occupational attainment, BMI, memory self-satisfaction, mental activity, physical activity, and social activity showed significant intergroup differences, and further analyses were conducted on these nine factors. Age, memory self-satisfaction, and mental activity were overlapping influencing factors of SCA and MCI. The results of specific items in the leisure activity scale are presented in Table S2.

**Table 1:** Significant intergroup differences in multidomain cognitive performance and behavioral characteristics of the three groups

Variables (M ± SD)	SCA (*n* = 154)	CNC (*n* = 173)	MCI (*n* = 256)	*F/ χ*^2^ /*H*	*P*-value
**General cognition**					
MMSE	28.45 ± 1.24	27.32 ± 1.62	26.40 ± 1.69	83.03	< 0.001^a,b,c^
**Memory**					
AVLT-N5	8.44 ± 1.78	4.35 ± 0.64	1.82 ± 2.04	755.57	< 0.001^a,b,c^
AVLT-N1N5	38.90 ± 7.11	24.51 ± 4.05	16.43 ± 6.69	639.23	< 0.001^a,b,c^
CFT delay	15.37 ± 6.64	11.52 ± 5.60	7.33 ± 5.61	89.05	< 0.001^a,b,c^
**Visuospatial ability**					
CFT copy	34.70 ± 1.67	33.81 ± 2.79	31.30 ± 6.14	32.68	< 0.001^a,b,c^
CDT	26.05 ± 3.38	24.05 ± 4.51	21.28 ± 6.31	42.82	< 0.001^a,b,c^
**Attention**					
SDMT	35.94 ± 10.63	28.31 ± 8.71	21.68 ± 8.92	110.12	< 0.001^a,b,c^
TMT-A	56.87 ± 21.21	65.65 ± 18.68	92.77 ± 39.16	81.19	< 0.001^a,b,c^
**Executive function**					
SCWT	41.88 ± 21.03	49.58 ± 20.43	54.89 ± 28.70	13.14	< 0.001^a,b^
TMT-B	148.88 ± 53.41	196.84 ± 19.86	275.60 ± 101.16	154.05	< 0.001^a,b,c^
**Demographic Information**					
Age	73.84 ± 3.78	74.80 ± 3.89	76.11 ± 4.15	16.49	< 0.001^a,b,c^
Gender (W%)	64.9%	50.9%	52.0%	8.30	0.016^a,b^
Education	13.60 ± 3.13	11.46 ± 3.53	10.61 ± 4.03	32.40	< 0.001^a,b^
Occupation	0.69 ± 0.09	0.65 ± 0.11	0.64 ± 0.11	14.79	< 0.001^a,b^
**Mental Health**					
Memory self-satisfaction	6.53 ± 2.06	6.25 ± 1.86	5.71 ± 2.24	18.54	< 0.001^a,b,c^
**Physical Health**					
BMI	23.98 ± 2.91	25.05 ± 3.52	24.41 ± 3.28	4.39	0.013^a^
**Leisure Activity**					
Mental Activity	64.54 ± 23.35	57.72 ± 23.79	50.89 ± 20.20	12.23	< 0.001^a,b,c^
Physical Activity	43.20 ± 14.80	41.31 ± 16.02	36.57 ± 14.21	7.23	0.001^b,c^
Social Activity	30.05 ± 14.72	26.53 ± 15.50	22.35 ± 12.13	10.07	< 0.001^b,c^

*Abbreviations*: *H* Kruskal-Wallis’ *H* value, *gender (W%)* Percentage of women in all participants, *MMSE* Mini-mental state examination, *AVLT-N5* Auditory verbal learning test long-time delayed recall, *AVLT-N1N5* Auditory verbal learning test total recall, *CFT* Rey-osterrieth complex figure test, *CDT* Clock-drawing test, *SDMT* Symbol digit modalities test, *TMT-A* Trail-making test part A, *SCWT* Stroop color-word test, *TMT-B* Trail-making test part B

Significance: ^a^significant difference between the SCA and CNC groups; ^b^significant difference between the SCA and MCI groups; ^c^significant difference between the CNC and MCI groups

### Relative importance rank of the identified factors

The multivariable linear regression analysis of the grouped sample revealed that 36.0% of the total variance in cognitive performance was explained by these nine identified factors. Relative importance analyses found that the level of education (explaining 43.9% of the total *R*^2^) was the most important factor, followed by age (20.6%), mental activity (12.1%), and occupational attainment (11.3%) (shown in Table 2). These top four factors explained 31.6% of the total cognitive variance. Four cognitive domains were explained by these nine identified factors in different proportions: the *R*^2^ was 0.289 for memory, 0.158 for visuospatial ability, 0.266 for attention, and 0.167 for executive function. The level of education, age, mental activity, and occupational attainment were the top four factors for all cognitive domains, except that memory self-satisfaction attained the fourth position in the memory domain. The importance rankings of these factors were similar when analyzing the full sample (shown in Table S3).

**Table 2:** Results of the relative importance of influencing factors to each cognitive domain in the grouped sample (*N* = 583)

Cognitive domain	Factors	Multiple linear regression		Regression relative importance (lmg)
		Standardized *β*	*P*-value	
Cognitive Z-score (*R*^2^ =0.360)	Education	0.41	< 0.001	43.9%
	Age	−0.29	< 0.001	20.6%
	Mental activity	0.26	0.007	12.1%
	Occupation	0.07	0.294	11.3%
	Social activity	−0.18	0.113	5.8%
	Physical activity	0.05	0.599	3.8%
	MS	0.04	0.415	2.0%
	Gender	0.03	0.609	0.4%
	BMI	0.01	0.880	0.2%
Memory Z-score (*R*^2^ =0.289)	Education	0.35	< 0.001	38.4%
	Age	−0.22	< 0.001	16.3%
	Mental activity	0.21	0.030	12.6%
	MS	0.14	0.008	9.2%
	Occupation	0.04	0.473	8.7%
	Social activity	−0.20	0.075	6.5%
	Physical activity	0.12	0.218	5.6%
	Gender	0.09	0.084	2.5%
	BMI	−0.01	0.819	0.3%
Visuospatial Z-score (*R*^2^ =0.158)	Education	0.35	< 0.001	63.6%
	Occupation	0.02	0.781	12.3%
	Age	−0.14	0.017	9.5%
	Mental activity	0.15	0.164	7.0%
	Social activity	−0.22	0.074	3.5%
	Physical activity	0.08	0.428	1.6%
	Gender	−0.04	0.535	1.4%
	MS	< 0.01	0.995	0.8%
	BMI	0.01	0.887	0.3%
Attention Z-score (*R*^2^ =0.266)	Education	0.31	< 0.001	36.3%
	Age	−0.31	< 0.001	32.7%
	Occupation	0.06	0.322	10.1%
	Mental activity	0.14	0.141	8.7%
	Social activity	0.03	0.771	6.7%
	Physical activity	−0.09	0.340	3.1%
	Gender	0.04	0.492	0.9%
	MS	0.01	0.895	0.9%
	BMI	−0.02	0.663	0.6%
Executive Z-score (*R*^2^ =0.167)	Education	0.19	0.006	26.8%
	Mental activity	0.28	0.009	24.2%
	Age	−0.18	0.002	18.5%
	Occupation	0.07	0.281	11.2%
	Social activity	−0.12	0.314	9.6%
	Physical activity	0.02	0.831	7.3%
	Gender	0.04	0.437	1.5%
	MS	−0.02	0.787	0.7%
	BMI	−0.01	0.923	0.2%

*Abbreviations*: *MS* Memory self-satisfaction, *BMI* Body mass index

### Assessment of relationships among early- and late-life factors and cognition

**Fig. 2:**
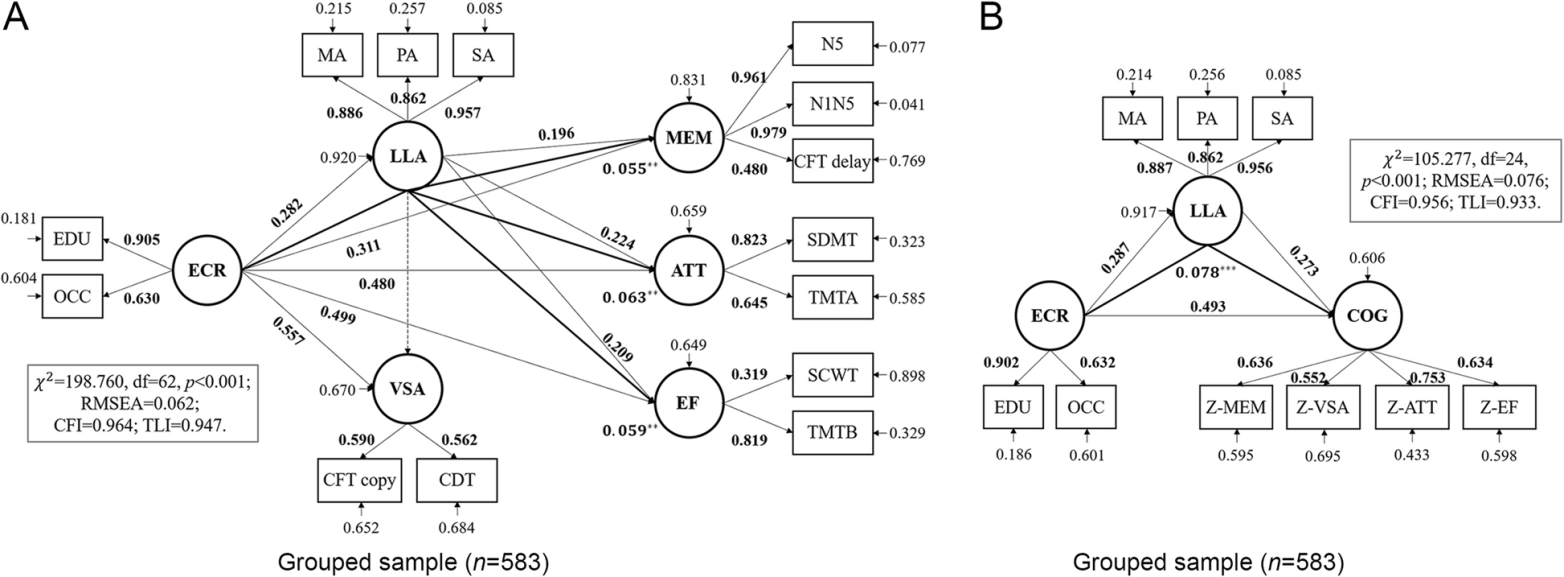
Structural equation models that reveal relationships among early-life cognitive reserve, late-life leisure activity, and cognitive performance. Full lines with arrows indicate significant paths and dotted lines indicate insignificant paths. Bold numbers without asterisks are significant path coefficients (*β*, all *p* < 0.001), bold numbers with asterisks (^*^p < 0.05, ^**^p < 0.01, and ^***^p < 0.001) indicate indirect effects, and narrow numbers are residual variances. Metrics that represent the goodness of model fit are listed separately. **A** Grouped sample (*N* = 583) SEM model built with early-life cognitive reserve, late-life leisure activity, and four cognitive domains. **B** Grouped sample (*N* = 583) SEM model built with early-life cognitive reserve, late-life leisure activity, and general cognitive function. Abbreviation: ECR, early-life cognitive reserve; LLA, late-life leisure activity; MEM, memory; VSA, visuospatial ability; ATT, attention; EF, executive function; EDU, level of education; OCC, occupational attainment; MA, mental activity; PA, physical activity; SA, social activity; N5, auditory verbal learning test long-time delayed recall; N1N5, auditory verbal learning test total recall; CFT, Rey-osterrieth complex figure test; CDT, clock-drawing test; SDMT, symbol digit modalities test; TMTA, trail-making test part A; SCWT, stroop color-word test; TMTB, trail-making test part B; COG, general cognitive function; Z-MEM, mean Z-score of tests in memory domain; Z-VSA, mean Z-score of tests in visuospatial ability domain; Z-ATT, mean Z-score of tests in attention domain; Z-EF, mean Z-score of tests in executive function domain

In our hypothesized model, the independent variable was a latent one (named early-life cognitive reserve, ECR) generated by educational and occupational attainment, and dependent variables were multidomain cognitive abilities including memory, visuospatial ability, attention, and executive function; moreover, a latent variable named as late-life leisure activity (LLA) was generated by mental, physical and social activities and was designed as the underlying mediating variable. In the grouped sample, the model fit indices were RMSEA = 0.062, CFI = 0.964, and TLI = 0.947, all of which indicated a good fit. Figure 2A shows the model of the grouped sample with standardized path coefficients, and a summary of all metrics of this model is provided in Table S4. LLA partially mediated the effects of ECR on memory, attention, and executive function, with indirect effects of 0.055, 0.063, and 0.059, corresponding to *p* = 0.002, 0.001, and 0.001, respectively. The model of the grouped sample with general cognitive function as dependent variable (latent variable generated by Z-scores of the four domains) also fitted well (RMSEA = 0.076, CFI = 0.956, and TLI = 0.933), and the partial mediating effect of LLA was significant (estimated mediating effect was 0.078, *p* < 0.001, shown in Fig. 2B and Table S5). The aforementioned models and results were also validated in the full sample (*N* = 1347), please see Fig. S1, Tables S6 and S7.

**Fig. 3:**
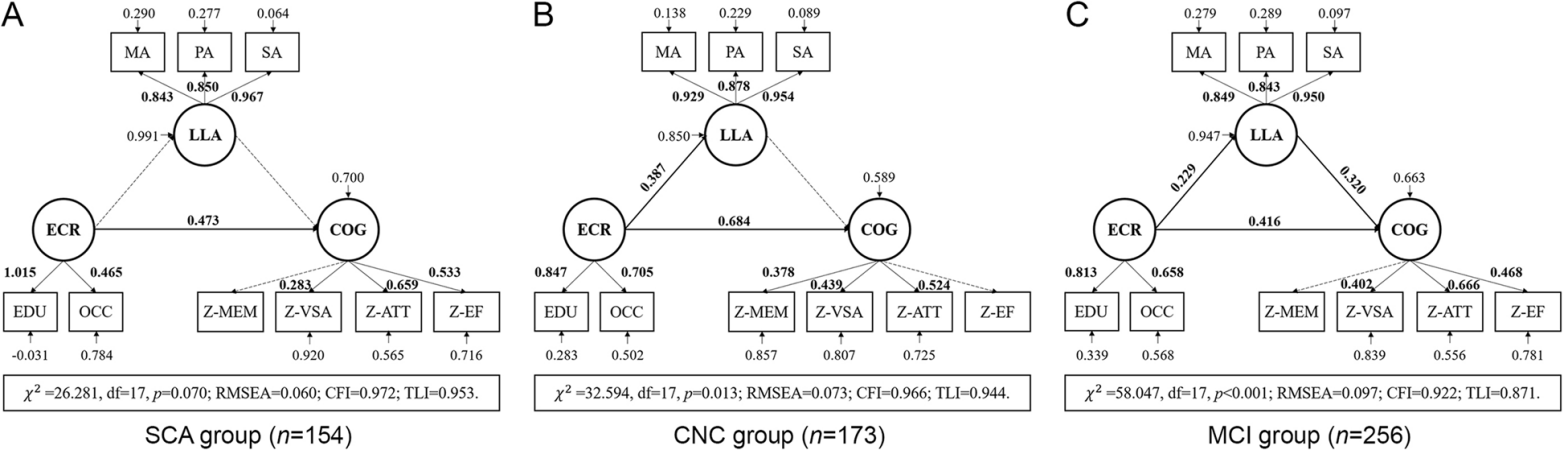
Intergroup trend of differences in structural equation models for different groups. Full lines indicate significant paths and dotted lines indicate insignificant paths. Bold numbers without asterisks are significant path coefficients (*β*, all *p* < 0.05), bold numbers with asterisks (^*^p < 0.05, ^**^p < 0.01, and ^***^p < 0.001) indicate indirect effects, and narrow numbers are residual variances. Metrics that represent the goodness of model fit are listed separately. **A** SEM model of the SCA group (*N* = 154); **B** SEM model of the CNC group (*N* = 173); **C** SEM model of the MCI group (*N* = 256). Abbreviation: ECR, early-life cognitive reserve; LLA, late-life leisure activity; COG, general cognitive function; EDU, level of education; OCC, occupational attainment; MA, mental activity; PA, physical activity; SA, social activity; Z-MEM, mean Z-score of tests in memory domain; Z-VSA, mean Z-score of tests in visuospatial ability domain; Z-ATT, mean Z-score of tests in attention domain; Z-EF, mean Z-score of tests in executive function domain

SEM models were further built for each of the three groups separately, with general cognitive function as the dependent variable. In the SCA group, the model fit indices were RMSEA = 0.060, CFI = 0.972, and TLI = 0.953, and the path from ECR to cognition was the only significant path in this model (shown in Fig. 3A and Table S8). In the CNC group, the model also displayed a good fit (RMSEA = 0.073, CFI = 0.966, and TLI = 0.944), and paths from ECR to both LLA and cognition were significant (shown in Fig. 3B and Table S8). In the MCI group, three paths between ECR, LLA, and general cognitive function were all significant, although this model fit less satisfactorily (RMSEA = 0.097, CFI = 0.922, and TLI = 0.871) (shown in Fig. 3C and Table S8). The mediating effect of LLA was insignificant in all three group-specific models listed above (SCA model, standardized *β* = 0.022, *p* = 0.440; CNC model, standardized *β* = − 0.057, *p* = 0.564; MCI model, standardized *β* = 0.073, *p* = 0.096). A tendency of differences in the effects of ECR and LLA on cognition in SCA and MCI groups was found: ECR, rather than LLA, exerted a pivotal and direct effect on the superior cognitive performance of the SCA group; but for individuals with MCI, both LLA and ECR played considerable roles on determining their cognition.

## Discussion

The current study showed that abundant early-life cognitive reserve and reduced late-life leisure activity were distinct characteristics of SCA and MCI, respectively. Common characteristics contributing to both aging processes were age, memory self-satisfaction, and mental activity. The level of education, age, mental activity, and occupational attainment were the top four important factors explaining 31.6% of cognitive aging heterogeneity. The results of SEM analyses showed that both early-life cognitive reserve and late-life leisure activity exerted direct effects on multiple cognitive domains, and late-life leisure activity partially mediated the relationship between early-life cognitive reserve and cognitive performance.

Our results suggested that a high level of early-life cognitive reserve played a central role in SCA by identifying that early-life educational and occupational attainment were specific factors influencing SCA. These two factors ranked in the top four in terms of importance in predicting cognitive performance, and early-life cognitive reserve exerted a significant and steady direct effect on cognitive performance in SEM models built in the grouped sample, the full sample, and the SCA group. This result is consistent with a previous study showing that SCA is determined by cognitive reserve acquired throughout life and mainly developed during the years of formal education [44]. In addition to early-life cognitive reserve, female sex and low BMI were specific characteristics of SCA. Previous studies have provided evidence supporting this female superiority in the longitudinal maintenance of cognitive function [5, 6]. The potential effect of obesity on cognitive aging is still controversial [45]; the low BMI characteristic of the SCA group found in this study requires further examination. We found that the MCI group tended to be inactive in daily life, and this result supported previous studies showing that interventions upregulating older adults’ multi-domain activity exerted positive effects on preventing pathological aging [18, 46]. Furthermore, this study implied previous work might have confused the group difference by including SCA older adults in the non-pathological aging group together with CNC. For example, we found educational differences between the SCA and CNC groups, but not between the MCI and CNC groups, although the level of education showed a group difference between pathological and non-pathological aging older adults [47].

Age, mental activity and memory self-satisfaction were overlapping factors contributing to both successful and pathological cognitive aging. Advanced age is undoubtedly an obvious risk factor for cognitive decline. Memory self-satisfaction reflects older adults’ subjective cognitive evaluation, which is valuable for the early diagnosis of subjective cognitive decline (SCD) [48]. In this study, regression analyses revealed this subjective evaluation significantly predicted objective memory performance (*β* =0.14, *p* = 0.008, lmg = 9.2%, shown in Table 2). Various types of intellectual engagements serve as proxy measures of cognitive reserve [49, 50], which continues to accumulate during the lifespan and in old age by participating in mental activities and cognitive training [46]. This study identified four pivotal factors that explained 31.6% of cognitive aging heterogeneity, and among them, the level of education, occupational attainment, and mental activity were all proxies of cognitive reserve [50], or intellectual enrichment. We propose that interventions aiming to upregulate lifetime intellectual enrichment would be effective measures for promoting successful cognitive aging.

The results of SEM analyses indicated that early-life cognitive reserve had a long-lasting influence on late-life leisure activity and subsequent cognitive performance. SEM analyses of different groups further revealed that early-life cognitive reserve played a pivotal role in the SCA group. The high early-life cognitive reserve may be the driving force for the maintenance of cognitive function in old age in the SCA group, which supports the “preserved differentiation” theory of how an influencing factor may operate concerning cognitive aging [1]. Nonetheless, a longitudinal study is necessary to further confirm the effect of early-life cognitive reserve on cognitive maintenance and decline throughout the lifespan. Because of the small sample size and limited cognitive variabilities within each group, the mediating effects of late-life leisure activity were insignificant in multi-group SEM analyses; on the other hand, the identified factors played more important roles in directing the different aging paths of participants than in explaining the variation of participants’ cognitive performance within each of the three groups.

The current study has some limitations. First, MCI cannot represent all behavioral features of pathological cognitive aging processes, but only typical ones of them; and there is more variance in pathological cognitive aging individuals (for example, dementia patients) that needs further investigation. Second, a consensus on the definition of SCA has still not been reached, previous studies have used different cognitive measures to define SCA, and we selected two high-profile domains (episodic memory and executive function); however, the generalization of the results of this study to other models of SCA should be performed with caution. Third, the current study only paid attention to the nine characteristics of interest and didn’t evaluate the underlying effects of other factors on cognition, resulting that nearly two-thirds of the total variance not being explained by the current regression algorithm and potential biases may exist, future studies should further explore other factors and their relative importance. In addition, although the current study tried to clarify the relationships between early-life and late-life influencing factors using SEM analyses, only longitudinal data will be capable of revealing the determinants of cognitive aging throughout life.

## Conclusions

This study found that early-life cognitive reserve and late-life leisure activity were specific characteristics of successful and pathological cognitive aging, separately, and suggested that measures upregulating lifetime intellectual enrichment would be beneficial to late-life cognitive performance.

## Supplementary Information

**Additional file 1: Table S1.** Intergroup differences in demographics, health conditions, and living habits. **Table S2.** Intergroup differences in detailed items of leisure activities. **Table S3.** Results of the relative importance of influencing factors to each cognitive domain in the full sample (*N* = 1347). **Table S4.** Estimated direct and indirect effects of the grouped sample (*N* = 583) SEM with ECR, LLA, and four cognitive domains as latent factors. **Table S5.** Estimated direct and indirect effects of the grouped sample (*N* = 583) SEM with ECR, LLA, and general cognitive function as latent factors. **Table S6.** Estimated direct and indirect effects of the full sample (*N* = 1347) SEM with ECR, LLA, and four cognitive domains as latent factors. **Table S7.** Estimated direct and indirect effects of the full sample (*N* = 1347) SEM with ECR, LLA, and general cognitive function as latent factors. **Table S8.** Estimated direct and indirect effects of the multi-group SEM with ECR, LLA, and general cognitive function as latent factors. **Figure S1.** Structural equation models that reveal relationships among ECR, LLA, and cognitive performance in the full sample (*N* = 1347).

## Data Availability

The dataset analyzed during the current study are not publicly available as these might contain information that could compromise the privacy of research participants, and the BABRI database continues to be constructed in an ongoing cohort study. Data analyzed in the current study are available from the corresponding author upon reasonable request.

## Abbreviations

SCA: Successful cognitive aging
MCI: Mild cognitive impairment
CNC: Cognitively normal control
MMSE: Mini-mental state examination
AVLT-N5: Auditory verbal learning test long-time delayed recall
TMT-B: Trail-making test part B
BMI: Body mass index
SEM: Structural equation model
RMSEA: Root mean square error of approximation
CFI: Comparative fit index
TLI: Tucker-Lewis index
ECR: Early-life cognitive reserve
LLA: Late-life leisure activity
