# Supplementary material for: Contributions of early-life cognitive reserve and late-life leisure activity to successful and pathological cognitive aging
Source: BMC Geriatr. 2022 Nov 1;22:831. doi: 10.1186/s12877-022-03530-5 (PMC9628084; doi:10.1186/s12877-022-03530-5)
Supplement: Supplementary file 1 — Additional file 1: Table S1. Intergroup differences in demographics, health conditions, and living habits. Table S2. Intergroup differences in detailed items of leisure activities. Table S3. Results of the relative importance of influencing factors to each cognitive domain in the full sample (N = 1347). Table S4. Estimated direct and indirect effects of the grouped sample (N = 583) SEM with ECR, LLA, and four cognitive domains as latent factors. Table S5. Estimated direct and indirect effects of the grouped sample (N = 583) SEM with ECR, LLA, and general cognitive function as latent factors. Table S6. Estimated direct and indirect effects of the full sample (N = 1347) SEM with ECR, LLA, and four cognitive domains as latent factors. Table S7. Estimated direct and indirect effects of the full sample (N = 1347) SEM with ECR, LLA, and general cognitive function as latent factors. Table S8. Estimated direct and indirect effects of the multi-group SEM with ECR, LLA, and general cognitive function as latent factors. Figure S1. Structural equation models that reveal relationships among ECR, LLA, and cognitive performance in the full sample (N = 1347). [file 12877_2022_3530_MOESM1_ESM.docx]

**Supplementary Materials**

**CONTENT**

- **Supplementary Tables**
- **Table S1.** Intergroup differences in demographics, health conditions, and living habits.
- **Table S2.** Intergroup differences in detailed items of leisure activities.
- **Table S3.** Results of the relative importance of influencing factors to each cognitive domain in the full sample (N=1347).
- **Table S4.** Estimated direct and indirect effects of the grouped sample (N=583) SEM with ECR, LLA, and four cognitive domains as latent factors.
- **Table S5.** Estimated direct and indirect effects of the grouped sample (N=583) SEM with ECR, LLA, and general cognitive function as latent factors.
- **Table S6.** Estimated direct and indirect effects of the full sample (N=1347) SEM with ECR, LLA, and four cognitive domains as latent factors.
- **Table S7.** Estimated direct and indirect effects of the full sample (N=1347) SEM with ECR, LLA, and general cognitive function as latent factors.
- **Table S8.** Estimated direct and indirect effects of the multi-group SEM with ECR, LLA, and general cognitive function as latent factors.
- **Supplementary Figures**
- **Figure S1.** Structural equation models that reveal relationships among ECR, LLA, and cognitive performance in the full sample (N=1347).

**Supplementary Tables**

**Table S1. Intergroup differences in demographics, health conditions, and living habits.**

| Variables (M±SD) | SCA (n=154) | CNC (n=173) | MCI (n=256) | *F*/$\chi^{2}$/*H* | *p*-value |
| --- | --- | --- | --- | --- | --- |
| **Demographic Information** | | | | | |
| Marriage (Y/N) | 126/25 | 143/28 | 188/54 | 3.08 | 0.214 |
| Income | 8.92±3.03 | 8.87±2.81 | 8.53±2.92 | 1.25 | 0.535 |
| **Mental Health** | | | | | |
| Life self-satisfaction | 5.60±1.08 | 5.35±1.11 | 5.33±1.18 | 5.60 | 0.061 |
| Loneliness | 32.75±7.69 | 32.51±8.44 | 34.56±9.33 | 2.08 | 0.127 |
| Depression | 6.38±5.28 | 6.96±5.75 | 7.82±6.13 | 2.47 | 0.086 |
| **Physical Health** | | | | | |
| Self-evaluation | 3.28±0.65 | 3.17±0.64 | 3.08±0.72 | 7.10 | 0.029^b^ |
| Diabetes | 22.7% | 26.2% | 31.1% | 3.53 | 0.171 |
| Hypertension | 54.6% | 65.7% | 62.5% | 4.51 | 0.105 |
| Hyperlipidemia | 47.9% | 43.3% | 40.3% | 2.12 | 0.347 |
| CVD | 24.0% | 22.8% | 27.5% | 1.33 | 0.514 |
| CHD | 22.8% | 27.1% | 30.3% | 2.47 | 0.290 |
| **Living habit** | | | | | |
| Eating regularity | 4.90±2.44 | 4.72±3.12 | 5.29±2.51 | 3.90 | 0.142 |
| Sleep regularity | 4.64±2.39 | 4.30±2.74 | 4.89±2.43 | 2.95 | 0.228 |
| Smoking | 16.9% | 23.4% | 17.3% | 2.68 | 0.262 |
| Drinking | 18.5% | 19.7% | 22.2% | 0.66 | 0.720 |
| PSQI Score | 6.57±3.26 | 6.69±3.93 | 6.99±4.18 | 0.21 | 0.815 |

*Note.* Abbreviation: marriage (Y: yes, married; N: no, single for various reasons including unmarried, widowed, and divorced); CVD, cerebrovascular disease; CHD, coronary heart disease; PSQI, Pittsburgh Sleep Quality Index; *H*, Kruskal-Wallis’ *H* value. Significance: ^a^ significant difference between the SCA and CNC groups; ^b^ significant difference between the SCA and MCI groups; ^c^ significant difference between the CNC and MCI groups.

**Table S2. Intergroup differences in detailed items of leisure activities.**

| Factors | SCA (n=154) | CNC (n=173) | MCI (n=256) | *H* value | *p-*value |
| --- | --- | --- | --- | --- | --- |
| Reading | 3.37±1.21 | 3.06±1.52 | 2.87±1.65 | 6.29 | 0.043^b^ |
| Writing | 0.75±1.29 | 0.49±1.07 | 0.49±1.20 | 8.73 | 0.013^b^ |
| Course learning | 0.68±1.10 | 0.35±0.88 | 0.33±0.90 | 16.59 | <0.001^a,b^ |
| Table games | 0.88±1.36 | 0.86±1.35 | 0.63±1.26 | 5.50 | 0.064 |
| Handwork | 0.67±1.30 | 0.31±0.84 | 0.10±0.43 | 24.85 | <0.001^a,b,c^ |
| Calligraphy | 0.92±1.32 | 0.82±1.34 | 0.57±1.20 | 9.35 | 0.009^b^ |
| Arts | 0.99±1.45 | 0.78±1.34 | 0.62±1.27 | 5.31 | 0.070 |
| Watching TV | 3.88±0.67 | 3.90±0.52 | 3.92±0.45 | 0.25 | 0.883 |
| Playing computers | 2.00±1.87 | 1.67±1.92 | 1.10±1.75 | 19.88 | <0.001^b,c^ |
| Playing puzzles | 0.94±1.51 | 0.31±0.95 | 0.27±0.91 | 33.16 | <0.001^a,b^ |
| Exercise | 3.58±1.07 | 3.58±1.11 | 3.36±1.36 | 3.06 | 0.216 |
| Fitness | 0.26±0.83 | 0.43±1.10 | 0.23±0.86 | 4.10 | 0.129 |
| Agility | 1.17±1.70 | 1.16±1.73 | 0.92±1.61 | 2.84 | 0.242 |
| Taiji | 0.59±1.32 | 0.52±1.24 | 0.39±1.11 | 2.93 | 0.231 |
| Outdoor activities | 0.67±1.02 | 0.51±1.05 | 0.47±0.98 | 7.44 | 0.024^a,b^ |
| Travel | 0.71±0.59 | 0.64±0.59 | 0.53±0.57 | 8.27 | 0.016^b^ |
| Team games | 0.16±0.58 | 0.19±0.70 | 0.10±0.50 | 3.04 | 0.218 |
| Planting | 2.50±1.83 | 2.58±1.83 | 2.51±1.88 | 0.22 | 0.896 |
| Pet | 0.84±1.62 | 0.93±1.69 | 0.92±1.68 | 0.23 | 0.890 |
| Visiting relatives | 1.73±0.98 | 1.44±0.91 | 1.40±0.93 | 11.22 | 0.004^a,b^ |
| Party | 0.89±0.99 | 0.78±0.90 | 0.80±0.93 | 0.73 | 0.694 |
| Doing housework | 3.96±0.24 | 3.84±0.74 | 3.61±1.09 | 14.44 | 0.001^b,c^ |
| Babysitting | 1.39±1.79 | 1.07±1.69 | 0.84±1.57 | 9.11 | 0.011^b^ |

*Note.* Significance: ^a^ significant difference between the SCA and CNC groups; ^b^ significant difference between the SCA and MCI groups; ^c^ significant difference between the CNC and MCI groups. *H*, Kruskal-Wallis’ *H* value.

**Table S3. Results of the relative importance of influencing factors to each cognitive domain in the full sample (N=1347).**

| Cognitive domain | Factors | Multiple linear regression | | Regression relative importance (lmg) |
| --- | --- | --- | --- | --- |
|  |  | Standardized *β* | *p*-value |  |
| Cognitive Z-score  ($R^{2}$=0.326) | Education | 0.38 | <0.001 | 43.3% |
|  | Age | -0.27 | <0.001 | 19.5% |
|  | Mental activity | 0.25 | <0.001 | 12.9% |
|  | Occupation | 0.08 | 0.065 | 12.8% |
|  | Social activity | -0.12 | 0.140 | 6.2% |
|  | Physical activity | -0.01 | 0.917 | 3.7% |
|  | MS | 0.03 | 0.392 | 0.9% |
|  | BMI | -0.02 | 0.594 | 0.4% |
|  | Gender | 0.01 | 0.750 | 0.4% |
| Memory Z-score  ($R^{2}$=0.195) | Education | 0.28 | <0.001 | 37.1% |
|  | Age | -0.18 | <0.001 | 15.3% |
|  | MS | 0.15 | <0.001 | 13.0% |
|  | Mental activity | 0.17 | 0.020 | 12.5% |
|  | Occupation | 0.02 | 0.588 | 7.9% |
|  | Social activity | -0.13 | 0.130 | 6.3% |
|  | Physical activity | 0.06 | 0.403 | 5.1% |
|  | Gender | 0.08 | 0.025 | 2.5% |
|  | BMI | -0.02 | 0.626 | 0.4% |
| Visuospatial Z-score  ($R^{2}$=0.152) | Education | 0.31 | <0.001 | 57.7% |
|  | Occupation | 0.05 | 0.303 | 15.5% |
|  | Age | -0.13 | 0.001 | 8.8% |
|  | Mental activity | 0.14 | 0.061 | 7.2% |
|  | Gender | -0.07 | 0.063 | 4.7% |
|  | Social activity | -0.18 | 0.035 | 3.1% |
|  | Physical activity | 0.05 | 0.462 | 1.4% |
|  | BMI | -0.03 | 0.476 | 1.3% |
|  | MS | -0.03 | 0.492 | 0.3% |
| Attention Z-score ($R^{2}$=0.270) | Education | 0.32 | <0.001 | 41.5% |
|  | Age | -0.25 | <0.001 | 20.3% |
|  | Occupation | 0.10 | 0.020 | 15.2% |
|  | Mental activity | 0.20 | 0.005 | 12.1% |
|  | Social activity | -0.03 | 0.670 | 6.6% |
|  | Physical activity | -0.05 | 0.454 | 3.5% |
|  | Gender | 0.01 | 0.866 | 0.4% |
|  | MS | -0.01 | 0.829 | 0.3% |
|  | BMI | <0.01 | 0.926 | 0.1% |
| Executive Z-score ($R^{2}$=0.155) | Age | -0.20 | 0.001 | 23.6% |
|  | Education | 0.15 | 0.001 | 22.7% |
|  | Mental activity | 0.22 | 0.003 | 20.2% |
|  | Occupation | 0.09 | 0.058 | 12.0% |
|  | Social activity | 0.02 | 0.847 | 10.9% |
|  | Physical activity | -0.08 | 0.262 | 6.0% |
|  | BMI | -0.06 | 0.127 | 2.6% |
|  | Gender | 0.05 | 0.158 | 1.5% |
|  | MS | -0.02 | 0.520 | 0.5% |

*Note.* Abbreviation: MS: memory self-satisfaction; BMI: body mass index.

**Table S4. Estimated direct and indirect effects of the grouped sample (N=583) SEM with ECR, LLA, and four cognitive domains as latent factors.**

| Model paths | Estimate | S.E. | Est./S.E. | *p*-value | 95%CI |
| --- | --- | --- | --- | --- | --- |
| **Measurement model** | | | | | |
| ECR$\to$EDU | 0.905 | 0.047 | 19.386 | <0.001 | (0.813, 0.996) |
| ECR$\to$OCC | 0.630 | 0.040 | 15.719 | <0.001 | (0.551, 0.708) |
| LLA$\to$MA | 0.886 | 0.015 | 59.079 | <0.001 | (0.856, 0.915) |
| LLA$\to$PA | 0.862 | 0.016 | 52.390 | <0.001 | (0.830, 0.894) |
| LLA$\to$SA | 0.957 | 0.012 | 82.245 | <0.001 | (0.934, 0.979) |
| MEM$\to$N5 | 0.961 | 0.008 | 115.292 | <0.001 | (0.944, 0.977) |
| MEM$\to$N1N5 | 0.979 | 0.009 | 111.542 | <0.001 | (0.962, 0.996) |
| MEM$\to$CFT delay | 0.480 | 0.035 | 13.911 | <0.001 | (0.413, 0.548) |
| VSA$\to$CFT copy | 0.590 | 0.056 | 10.472 | <0.001 | (0.479, 0.700) |
| VSA$\to$CDT | 0.562 | 0.051 | 11.023 | <0.001 | (0.462, 0.662) |
| ATT$\to$SDMT | 0.823 | 0.026 | 31.206 | <0.001 | (0.771, 0.874) |
| ATT$\to$TMTA | 0.645 | 0.030 | 21.709 | <0.001 | (0.586, 0.703) |
| EF$\to$SCWT | 0.319 | 0.046 | 6.922 | <0.001 | (0.229, 0.410) |
| EF$\to$TMTB | 0.819 | 0.057 | 14.475 | <0.001 | (0.708, 0.930) |
| **Structural model: direct effects** | | | | | |
| ECR$\to$LLA | 0.282 | 0.055 | 5.134 | <0.001 | (0.174, 0.390) |
| LLA$\to$MEM | 0.196 | 0.052 | 3.739 | <0.001 | (0.093, 0.299) |
| LLA$\to$VSA | 0.054 | 0.073 | 0.745 | 0.456 | (-0.089, 0.198) |
| LLA$\to$ATT | 0.224 | 0.060 | 3.716 | <0.001 | (0.106, 0.343) |
| LLA$\to$EF | 0.209 | 0.058 | 3.577 | <0.001 | (0.094, 0.323) |
| ECR$\to$MEM | 0.311 | 0.043 | 7.159 | <0.001 | (0.226, 0.396) |
| ECR$\to$VSA | 0.557 | 0.068 | 8.163 | <0.001 | (0.423, 0.691) |
| ECR$\to$ATT | 0.480 | 0.055 | 8.749 | <0.001 | (0.372, 0.587) |
| ECR$\to$EF | 0.499 | 0.069 | 7.225 | <0.001 | (0.364, 0.634) |
| **Structural model: indirect effects** | | | | | |
| ECR$\to$LLA$\to$MEM | 0.055 | 0.018 | 3.102 | 0.002 | (0.020, 0.090) |
| ECR$\to$LLA$\to$VSA | 0.015 | 0.021 | 0.735 | 0.463 | (-0.026, 0.056) |
| ECR$\to$LLA$\to$ATT | 0.063 | 0.020 | 3.217 | 0.001 | (0.025, 0.102) |
| ECR$\to$LLA$\to$EF | 0.059 | 0.018 | 3.201 | 0.001 | (0.023, 0.095) |
| **Structural model: total effect** | | | | | |
| ECR$\to$MEM | 0.366 | 0.040 | 9.050 | <0.001 | (0.287, 0.445) |
| ECR$\to$VSA | 0.572 | 0.063 | 9.113 | <0.001 | (0.449, 0.696) |
| ECR$\to$ATT | 0.543 | 0.050 | 10.840 | <0.001 | (0.445, 0.641) |
| ECR$\to$EF | 0.558 | 0.067 | 8.318 | <0.001 | (0.426, 0.689) |

*Note.* Abbreviation: S.E. standard error; Est.: estimate; CI: confidence interval; ECR, early-life cognitive reserve; LLA, late-life leisure activity; EDU, level of education; OCC, occupational attainment; MA, mental activity; PA, physical activity; SA, social activity; MEM, memory; VSA, visuospatial ability; ATT, attention; EF, executive function; N5, auditory verbal learning test long-time delayed recall; N1N5, auditory verbal learning test total recall; CFT delay, Rey-Osterrieth complex figure test recall; CFT copy, Rey-Osterrieth complex figure test copy; CDT, clock-drawing test; SDMT, symbol digit modalities test; TMTA, trail-making test part A; SCWT, symbol digit modalities test; TMTB, trail-making test part B.

**Table S5. Estimated direct and indirect effects of the grouped sample (N=583) SEM with ECR, LLA, and general cognitive function as latent factors.**

| Model paths | Estimate | S.E. | Est./S.E. | *p*-value | 95%CI |
| --- | --- | --- | --- | --- | --- |
| **Measurement model** | | | | | |
| ECR$\to$EDU | 0.902 | 0.054 | 16.669 | <0.001 | (0.796, 1.008) |
| ECR$\to$OCC | 0.632 | 0.044 | 14.519 | <0.001 | (0.546, 0.717) |
| LLA$\to$MA | 0.887 | 0.015 | 59.498 | <0.001 | (0.857, 0.916) |
| LLA$\to$PA | 0.862 | 0.016 | 52.690 | <0.001 | (0.830, 0.894) |
| LLA$\to$SA | 0.956 | 0.012 | 83.066 | <0.001 | (0.934, 0.979) |
| COG$\to$Z-MEM | 0.636 | 0.034 | 18.672 | <0.001 | (0.569, 0.703) |
| COG$\to$Z-VSA | 0.552 | 0.037 | 14.831 | <0.001 | (0.479, 0.625) |
| COG$\to$Z-ATT | 0.753 | 0.029 | 26.272 | <0.001 | (0.697, 0.809) |
| COG$\to$Z-EF | 0.634 | 0.037 | 17.022 | <0.001 | (0.561, 0.707) |
| **Structural model: direct effects** | | | | | |
| ECR$\to$LLA | 0.287 | 0.056 | 5.139 | <0.001 | (0.178, 0.397) |
| LLA$\to$COG | 0.273 | 0.057 | 4.768 | <0.001 | (0.161, 0.385) |
| ECR$\to$COG | 0.493 | 0.057 | 8.628 | <0.001 | (0.381, 0.604) |
| **Structural model: indirect effects** | | | | | |
| ECR$\to$LLA$\to$COG | 0.078 | 0.021 | 3.774 | <0.001 | (0.038, 0.119) |
| **Structural model: total effect** | | | | | |
| ECR$\to$COG | 0.571 | 0.053 | 10.860 | <0.001 | (0.468, 0.674) |

*Note.* Abbreviation: S.E. standard error; Est.: estimate; CI: confidence interval; ECR, early-life cognitive reserve; LLA, late-life leisure activity; EDU, level of education; OCC, occupational attainment; MA, mental activity; PA, physical activity; SA, social activity; Z-MEM, mean Z-score of tests in memory domain; Z-VSA, mean Z-score of tests in visuospatial ability domain; Z-ATT, mean Z-score of tests in attention domain; Z-EF, mean Z-score of tests in executive function domain; COG, general cognitive function.

**Table S6. Estimated direct and indirect effects of the full sample (N=1347) SEM with ECR, LLA, and four cognitive domains as latent factors.**

| Model paths | Estimate | S.E. | Est./S.E. | *p*-value | 95%CI |
| --- | --- | --- | --- | --- | --- |
| **Measurement model** | | | | | |
| ECR$\to$EDU | 0.916 | 0.030 | 30.556 | <0.001 | (0.857, 0.974) |
| ECR$\to$OCC | 0.607 | 0.027 | 22.561 | <0.001 | (0.555, 0.660) |
| LLA$\to$MA | 0.884 | 0.009 | 96.346 | <0.001 | (0.866, 0.902) |
| LLA$\to$PA | 0.855 | 0.011 | 80.922 | <0.001 | (0.835, 0.876) |
| LLA$\to$SA | 0.964 | 0.007 | 140.786 | <0.001 | (0.950, 0.977) |
| MEM$\to$N5 | 0.928 | 0.010 | 90.548 | <0.001 | (0.908, 0.948) |
| MEM$\to$N1N5 | 0.971 | 0.010 | 98.168 | <0.001 | (0.952, 0.991) |
| MEM$\to$CFT delay | 0.325 | 0.026 | 12.425 | <0.001 | (0.274, 0.377) |
| VSA$\to$CFT copy | 0.480 | 0.040 | 12.104 | <0.001 | (0.402, 0.557) |
| VSA$\to$CDT | 0.450 | 0.039 | 11.540 | <0.001 | (0.374, 0.526) |
| ATT$\to$SDMT | 0.778 | 0.019 | 40.826 | <0.001 | (0.740, 0.815) |
| ATT$\to$TMTA | 0.659 | 0.020 | 32.389 | <0.001 | (0.619, 0.698) |
| EF$\to$SCWT | 0.296 | 0.030 | 9.910 | <0.001 | (0.238, 0.355) |
| EF$\to$TMTB | 0.792 | 0.042 | 19.070 | <0.001 | (0.711, 0.874) |
| **Structural model: direct effects** | | | | | |
| ECR$\to$LLA | 0.291 | 0.037 | 7.928 | <0.001 | (0.219, 0.363) |
| LLA$\to$MEM | 0.137 | 0.036 | 3.855 | <0.001 | (0.068, 0.207) |
| LLA$\to$VSA | 0.056 | 0.061 | 0.916 | 0.360 | (-0.063, 0.175) |
| LLA$\to$ATT | 0.198 | 0.042 | 4.730 | <0.001 | (0.116, 0.281) |
| LLA$\to$EF | 0.175 | 0.042 | 4.170 | <0.001 | (0.092, 0.257) |
| ECR$\to$MEM | 0.233 | 0.030 | 7.740 | <0.001 | (0.174, 0.292) |
| ECR$\to$VSA | 0.623 | 0.058 | 10.773 | <0.001 | (0.510, 0.737) |
| ECR$\to$ATT | 0.524 | 0.038 | 13.767 | <0.001 | (0.449, 0.599) |
| ECR$\to$EF | 0.520 | 0.046 | 11.375 | <0.001 | (0.430, 0.609) |
| **Structural model: indirect effects** | | | | | |
| ECR$\to$LLA$\to$MEM | 0.040 | 0.012 | 3.423 | 0.001 | (0.017, 0.063) |
| ECR$\to$LLA$\to$VSA | 0.016 | 0.018 | 0.915 | 0.360 | (-0.018, 0.051) |
| ECR$\to$LLA$\to$ATT | 0.058 | 0.013 | 4.372 | <0.001 | (0.032, 0.083) |
| ECR$\to$LLA$\to$EF | 0.051 | 0.012 | 4.069 | <0.001 | (0.026, 0.075) |
| **Structural model: total effect** | | | | | |
| ECR$\to$MEM | 0.273 | 0.027 | 9.956 | <0.001 | (0.220, 0.327) |
| ECR$\to$VSA | 0.640 | 0.054 | 11.929 | <0.001 | (0.534, 0.745) |
| ECR$\to$ATT | 0.582 | 0.034 | 17.075 | <0.001 | (0.515, 0.649) |
| ECR$\to$EF | 0.570 | 0.043 | 13.183 | <0.001 | (0.485, 0.655) |

*Note.* Abbreviation: S.E. standard error; Est.: estimate; CI: confidence interval; ECR, early-life cognitive reserve; LLA, late-life leisure activity; EDU, level of education; OCC, occupational attainment; MA, mental activity; PA, physical activity; SA, social activity; MEM, memory; VSA, visuospatial ability; ATT, attention; EF, executive function; N5, auditory verbal learning test long-time delayed recall; N1N5, auditory verbal learning test total recall; CFT delay, Rey-Osterrieth complex figure test recall; CFT copy, Rey-Osterrieth complex figure test copy; CDT, clock-drawing test; SDMT, symbol digit modalities test; TMTA, trail-making test part A; SCWT, symbol digit modalities test; TMTB, trail-making test part B.

**Table S7. Estimated direct and indirect effects of the full sample (N=1347) SEM with ECR, LLA, and general cognitive function as latent factors.**

| Model paths | Estimate | S.E. | Est./S.E. | *p*-value | 95%CI |
| --- | --- | --- | --- | --- | --- |
| **Measurement model** | | | | | |
| ECR$\to$EDU | 0.913 | 0.035 | 26.455 | <0.001 | (0.845, 0.981) |
| ECR$\to$OCC | 0.609 | 0.029 | 20.981 | <0.001 | (0.552, 0.666) |
| LLA$\to$MA | 0.885 | 0.009 | 96.683 | <0.001 | (0.867, 0.903) |
| LLA$\to$PA | 0.856 | 0.011 | 81.258 | <0.001 | (0.835, 0.877) |
| LLA$\to$SA | 0.963 | 0.007 | 141.604 | <0.001 | (0.950, 0.976) |
| COG$\to$Z-MEM | 0.539 | 0.027 | 19.697 | <0.001 | (0.486, 0.593) |
| COG$\to$Z-VSA | 0.504 | 0.029 | 17.587 | <0.001 | (0.448, 0.561) |
| COG$\to$Z-ATT | 0.790 | 0.022 | 36.072 | <0.001 | (0.747, 0.832) |
| COG$\to$Z-EF | 0.622 | 0.026 | 23.936 | <0.001 | (0.571, 0.672) |
| **Structural model: direct effects** | | | | | |
| ECR$\to$LLA | 0.294 | 0.037 | 7.975 | <0.001 | (0.221, 0.366) |
| LLA$\to$COG | 0.244 | 0.039 | 6.213 | <0.001 | (0.167, 0.321) |
| ECR$\to$COG | 0.506 | 0.039 | 13.068 | <0.001 | (0.430, 0.582) |
| **Structural model: indirect effects** | | | | | |
| ECR$\to$LLA$\to$COG | 0.072 | 0.013 | 5.311 | <0.001 | (0.045, 0.098) |
| **Structural model: total effect** | | | | | |
| ECR$\to$COG | 0.578 | 0.035 | 16.628 | <0.001 | (0.510, 0.646) |

*Note.* Abbreviation: S.E. standard error; Est.: estimate; CI: confidence interval; ECR, early-life cognitive reserve; LLA, late-life leisure activity; EDU, level of education; OCC, occupational attainment; MA, mental activity; PA, physical activity; SA, social activity; Z-MEM, mean Z-score of tests in memory domain; Z-VSA, mean Z-score of tests in visuospatial ability domain; Z-ATT, mean Z-score of tests in attention domain; Z-EF, mean Z-score of tests in executive function domain; COG, general cognitive function.

**Table S8. Estimated direct and indirect effects of the multi-group SEM with ECR, LLA, and general cognitive function as latent factors.**

|  | SCA group (N=154) | | | CNC group (N=173) | | | MCI group (N=256) | | |
| --- | --- | --- | --- | --- | --- | --- | --- | --- | --- |
| Model paths | Estimate (95%CI) | S.E. | *p*-value | Estimate (95%CI) | S.E. | *p*-value | Estimate (95%CI) | S.E. | *p*-value |
| **Measurement model** | | | | | | | | | |
| ECR$\to$EDU | 1.015 (0.080, 1.950) | 0.477 | 0.033 | 0.847 (0.639, 1.055) | 0.106 | <0.001 | 0.813 (0.408, 1.218) | 0.207 | <0.001 |
| ECR$\to$OCC | 0.465 (0.201, 0.728) | 0.134 | 0.001 | 0.705 (0.540, 0.870) | 0.084 | <0.001 | 0.658 (0.375, 0.940) | 0.144 | <0.001 |
| LLA$\to$MA | 0.843 (0.776, 0.909) | 0.034 | <0.001 | 0.929 (0.886, 0.971) | 0.022 | <0.001 | 0.849 (0.789, 0.909) | 0.030 | <0.001 |
| LLA$\to$PA | 0.850 (0.779, 0.922) | 0.036 | <0.001 | 0.878 (0.830, 0.926) | 0.024 | <0.001 | 0.843 (0.787, 0.900) | 0.029 | <0.001 |
| LLA$\to$SA | 0.967 (0.918, 1.017) | 0.025 | <0.001 | 0.954 (0.912, 0.997) | 0.022 | <0.001 | 0.950 (0.913, 0.988) | 0.019 | <0.001 |
| COG$\to$Z-MEM | n.s. | n.s. | n.s. | 0.378 (0.167, 0.589) | 0.108 | <0.001 | n.s. | n.s. | n.s. |
| COG$\to$Z-VSA | 0.283 (0.083, 0.483) | 0.102 | 0.006 | 0.439 (0.172, 0.706) | 0.136 | 0.001 | 0.402 (0.168, 0.636) | 0.119 | 0.001 |
| COG$\to$Z-ATT | 0.659 (0.438, 0.881) | 0.113 | <0.001 | 0.524 (0.273, 0.775) | 0.128 | <0.001 | 0.666 (0.392, 0.940) | 0.140 | <0.001 |
| COG$\to$Z-EF | 0.533 (0.294, 0.772) | 0.122 | <0.001 | n.s. | n.s. | n.s. | 0.468 (0.254, 0.682) | 0.109 | <0.001 |
| **Structural model: direct effects** | | | | | | | | | |
| ECR$\to$LLA | 0.093 (-0.093, 0.280) | 0.095 | 0.327 | 0.387 (0.155, 0.620) | 0.118 | 0.001 | 0.229 (0.015, 0.444) | 0.109 | 0.036 |
| LLA$\to$COG | 0.236 (-0.021, 0.494) | 0.131 | 0.071 | -0.148 (-0.577, 0.280) | 0.218 | 0.497 | 0.320 (0.072, 0.569) | 0.127 | 0.012 |
| ECR$\to$COG | 0.473 (0.192, 0.753) | 0.143 | 0.001 | 0.684 (0.348, 1.019) | 0.171 | <0.001 | 0.416 (0.105, 0.726) | 0.158 | 0.009 |
| **Structural model: indirect effects** | | | | | | | | | |
| ECR$\to$LLA$\to$COG | 0.022 (-0.034, 0.078) | 0.029 | 0.440 | -0.057 (-0.253, 0.138) | 0.100 | 0.564 | 0.073 (-0.013, 0.160) | 0.044 | 0.096 |
| **Structural model: total effect** | | | | | | | | | |
| ECR$\to$COG | 0.495 (0.217, 0.772) | 0.142 | <0.001 | 0.626 (0.365, 0.887) | 0.133 | <0.001 | 0.489 (0.188, 0.790) | 0.154 | 0.001 |

*Note*. Abbreviation: S.E. standard error; n.s.: not significant; ECR, early-life cognitive reserve; LLA, late-life leisure activity; EDU, level of education; OCC, occupational attainment; MA, mental activity; PA, physical activity; SA, social activity; Z-MEM, mean Z-score of tests in memory domain; Z-VSA, mean Z-score of tests in visuospatial ability domain; Z-ATT, mean Z-score of tests in attention domain; Z-EF, mean Z-score of tests in executive function domain; COG, general cognitive function.

**Supplementary Figures**


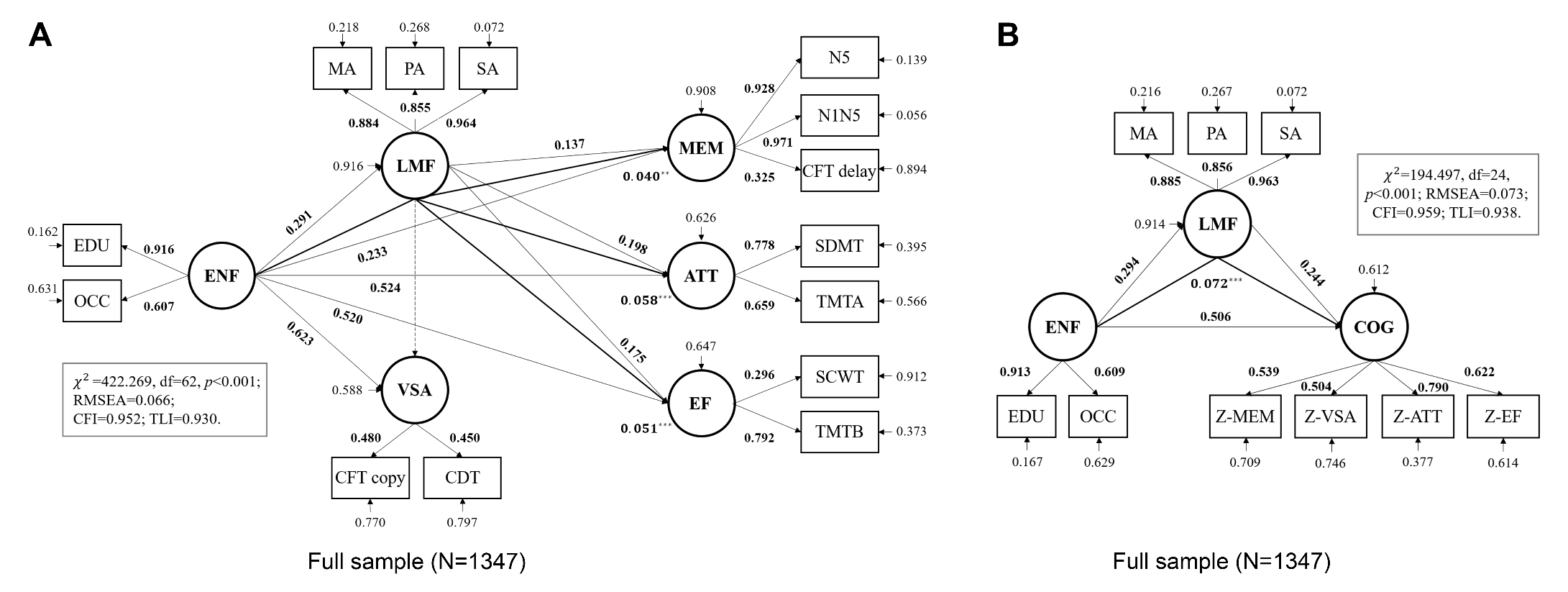


**Figure S1. Structural equation models that reveal relationships among ECR, LLA, and cognitive performance in the full sample (N=1347).** Full lines with arrows indicate significant paths and dotted lines indicate insignificant paths. Bold numbers without asterisks are significant path coefficients (*β*, all *p*<0.001), bold numbers with asterisks (^*^*p*<0.05, ^**^*p*<0.01, and ^***^*p*<0.001) indicate indirect effects, and narrow numbers are residual variances. Metrics that represent the goodness of model fit are listed separately. **(A)** Full sample (N=1347) SEM model built with ECR, LLA, and four cognitive domains. **(B)** Full sample (N=1347) SEM model built with ECR, LLA, and general cognitive function. Abbreviation: ECR, early-life cognitive reserve; LLA, late-life leisure activity; MEM, memory; VSA, visuospatial ability; ATT, attention; EF, executive function; EDU, level of education; OCC, occupational attainment; MA, mental activity; PA, physical activity; SA, social activity; N5, auditory verbal learning test long-time delayed recall; N1N5, auditory verbal learning test total recall; CFT, Rey-Osterrieth complex figure test; CDT, clock-drawing test; SDMT, symbol digit modalities test; TMTA, trail-making test part A; SCWT, Stroop color-word test; TMTB, trail-making test part B; COG, general cognitive function; Z-MEM, mean Z-score of tests in memory domain; Z-VSA, mean Z-score of tests in visuospatial ability domain; Z-ATT, mean Z-score of tests in attention domain; Z-EF, mean Z-score of tests in executive function domain.
